# Supplementary material for: The role of l-arabinose metabolism for Escherichia coli O157:H7 in edible plants
Source: Microbiology (Reading). 2021 Jul 28;167(7):001070. doi: 10.1099/mic.0.001070 (PMC8489885; doi:10.1099/mic.0.001070)
Supplement: Supplementary material 1 [file mic-167-1070-s001.pdf]

Supplementary Data & Text

Supplementary Text

P. atrosepticum **(Pba SCRI1043) transcriptional fusions**

A different vector plasmid was used to measure transcriptional activity of Pba (1043) *ara* genes because there were detrimental effects from the pACYC-based plasmid system. Growth of Pba (1043) was prolonged at 18 °C in RD MOPS glycerol compared to STEC (Sakai), with a cell density of only 0.68 (OD_{600}) after 24 h compared to ~2.8 for STEC (Sakai), necessitating expression analysis to be carried out at 27 °C where they reached a similar densities (~2.5). Although GFP expression was detectable from Pba (1043) transformed with either promoter fusion (pACYC*araBA*$_{\text{Pba}}$::*gfp*+, pJM064; pACYC*araF*$_{\text{Pba}}$::*gfp*+, pJM065), growth was inhibited at > 1mM L-Ara*p* (Supplementary Fig. 2G). GFP was induced, but the levels exceeded linear limits of detection for the plate reader (> 600,000 RFU). Therefore, the promoters for *araBA* and *araF* were sub-cloned into the low copy number pSC101-based plasmid, with ~ 6 copies / cell [1]. This strategy restored growth of the transformed Pba (1043) to that of the vector-only control (pWSK29) and the un-transformed strain (Supplementary Fig. 2G), even in the presence of 10 mM L-Ara*p*.

Supplementary Figure Legends

Supplementary Figure 1 Expression of *E. coli* O157:H7 *araA* in spinach and lettuce extracts

Expression of *araA* quantified by the DNA microarray, or by qPCR from the microarray samples (array) and independent repeated samples (repeat) in plant extracts (a), or following infiltration into leaves for one h (b). The culture controls were bacteria incubated in no-plant, vermiculite control, or in in the $MgCl_2$ infiltration medium. The average of three biological replicates, each with three technical replicates, with the SD.

Supplementary Figure 2 Growth curves of STEC (Sakai) and Pba (1043) transformed with fluorescent reporters

Fluorescent reporters pJM058 (A, B), pJM064 (C, D, G), pKC026 (E, F, G), pJM065, pJM067, pJM068 or pWSK029 (G) were transformed into STEC (Sakai WT) (A, C, E), STEC (Sakai Δ*araC*) (B, D, F), or Pba (1043) (G). Expression was monitored over time at 27 °C at arabinose concentrations of 0, 0.01, 0.1, 1 or 10 mM as indicated (A-F), or just at 10 mM (G).

Supplementary Figure 3 Genetic and structural organisation of the *ara* loci for STEC (Sakai) and Pba (1043).

Organisation of the genetic loci (a), with the metabolism genes in blue (*araB*, ribulokinase; *araA*, isomerase; *araD*, epimerase); transport genes in green (*araF*, arabinose binding protein; *araG*, ATP symporer; *araH*, permease); and regulator in orange (*araC*, binds L-arabinose). Gene clusters or genes that are in *cis* are marked by '//'. The STEC (Sakai) *araFGH* is on the complementary strand on the chromosome, and the CDS for *araH* is annotated as split between ECs2607 and ECs2606. Genetic distances are approximated on the gene clusters. Alignment of the AraC CDS (b) indicate the location of secondary structure features (as annotated for STEC Sakai).

Supplementary Figure 4 ***In planta* detection of GFP for controls and non-expressing constructs**

The empty GFP reporter vector, pKC026, was transformed in STEC (Sakai) or Pba (1043) to assess any background fluorescence. Epidermal and mesophyll cells of a leaf of *Nicotiana benthamiana* 4 days after infiltration with empty vector control pKC026 in Pba (a). Inoculated (seed imbibed) broccoli was harvested 6 days after germination for detection of plasmid-containing bacteria, treated with α-O157 primary antibody and labelled with Alexa Fluor® 568 nm secondary antibody (magenta) for detection on non-GFP STEC (Sakai). STEC WT transformed with pKC026 (b) or STEC Δ*araC* transformed $araF_{\mathrm{STEC}}$, $araBA_{\mathrm{Pba}}$ and $araF_{\mathrm{Pba}}$ constructs (c- f) were imaged on the epidermis with chloroplasts (blue) indicating the position of the epidermal and mesophyll cells. All scale bars represent 25 μm.

Supplementary Tables

Supplementary Table 1 Plasmids and primers used in the study

Plasmid / genotype	Gene / accession #	Vector	Cloning primers
pJM058<br>pACYC*araBAD*$_{STEC}$::*gfp*+	STEC (Sakai)<br>*araBAD*<br>ECs0067,<br>ECs0066,<br>ECs0065	pKC026	5'-GGTCTAGAGCCTGTCAAATGGACGAAGC<br>5'-CAGGTCGACTCTAGAGGATCC
pJM066<br>pACYC*araF*$_{STEC}$::*gfp*+	STEC (Sakai)<br>*araF*<br>ECs2609	pKC026	5'-GCTCTAGAGATGGCTCTCATTATACG<br>5'-CTCTAGAGCTTTAGTGTCGTTTTGTGCAGG
pJM064<br>pACYC*araBA*$_{Pba}$::*gfp*+	Pba (SRCI1043)<br>*araBA*<br>ECARS11170,<br>ECARS11175	pKC026	5'-GCTCTAGAGGTGTAATTATCTTTTCATTCGC<br>5'-GCTCTAGACTCATTAATCTCAGATCGTTGG
pJM065<br>pACYC*araF*$_{Pba}$::*gfp*+	Pba (SRCI1043)<br>*araF*<br>ECORS11165	pKC026	5'-GCTCTAGACTCATTAATCTCAGATCGTTGG<br>5'-GCTCTAGAGGTGTAATTATCTTTTCATTCGC
pJM067<br>pWSK*araBA*$_{Pba}$::*gfp*+	Pba (SRCI1043)<br>*araBA*<br>ECARS11170,<br>ECARS11175	pWSK29	5'-CCTGCAGGGTGTAATTATCTTTTCATTCGC<br>5'-GGCTGCAGGCCAGTTACCTCGGTTCAAA
pJM068<br>pWSK*araF*$_{Pba}$::*gfp*+	Pba (SRCI1043)<br>*araF*<br>ECORS11165	pWSK29	5'-GGCTGCAGGCCAGTTACCTCGGTTCAAA<br>5'-CCTGCAGGGTGTAATTATCTTTTCATTCGC
pTOF_ECs0066ko	ECs0066	pTOF24	ECs0066No_for: 5'-<br>AAAAACTGCAGCGGTCGGTCGATAAAAA<br>A<br>ECs0066Ni_rev: 5'-<br>CGCTTCTTGCGGCCGCTTGGAACGGAAA<br>GCTCGCACAGAATCA<br>ECs0066Ci-for: 5'-<br>CCGTTCCAAGCGGCCGCAAGAGCGGCC<br>AACTCACCGACTACT<br>ECs0066Co_rev: 5'-<br>AAAAAGTCGACCATTTGATTGGCTGTGGT<br>T
n/a	ECs0066	qRT-PCR	araA-F.1: 5'-CGGTCACTGGCAGGATAAAC<br>araA-R.1: 5'-GACGGAGAAACCGAACTTGA
n/a	ECs0065	qRT-PCR	araD-F.2: 5'-GCAAACGCTGCTGGATAAAC<br>araD-R.2: 5'-GCCTGGTTTCATTTGATTGG

Supplementary Table 2 Expression levels of arabinose reporters in xylose compared to arabinose

	Arabinose	Xylose	Xyl/Ara (%)	Glycerol	Gly/Ara (%)
**pJM058** pACYC*araBAD*$_{STEC}$::*gfp+*	2066 (± 62)	33 (± 6)	1.60	4 (±4)	0.20
**pJM066** pACYC*araF*$_{STEC}$::*gfp+*	1574 (±26)	528 (± 3)	33.53	151 (±14)	9.59
**pJM064** pACYC*araBA*$_{Pba}$::*gfp+*	14998 (±230)	2994 (±71)	19.96	1239 (±70)	8.26
**pJM065** pACYC*araF*$_{Pba}$::*gfp+*	31693 (±409)	13584 (±640)	42.86	8717 (±279)	27.50

GFP was measured in response to 10 mM L-arabinose, 10 mM D-xylose or no added sugar (i.e. glycerol) at maximal expression times (four or six h), for pJM058 or pJM066 transformed in STEC (Sakai) or pJM064 / pJM065 (multi-copy plasmids) in Pba (1043), at 27 °C. The proportion of expression relative to arabinose is shown for xylose and glycerol as a percentage.

Supplementary Table 3 **Antibody screening of a panel of plant species, cultivars and tissues**

				Averaged absorbance for antibodies (OD 405nm)						Standard deviation					
**Species name**	**Common name**	**Cultivar**	**Tissue**	**LM6**	**LM13**	**LM5**	**LM16**	**LM1**	**JIM13**	**LM6**	**LM13**	**LM5**	**LM16**	**LM1**	**JIM13**
Hordeum vulgare	barley	Optic	leaf	0.172	0.015	0.012	0.019	0.032	0.050	0.002	0.009	0.003	0.003	0.003	0.005
Ocimum basilicum	basil	Gecofure	leaf	0.080	0.003	0.039	0.009	0.002	0.365	0.002	0.001	0.010	0.005	0.001	0.008
Lactuca sativa	lettuce	All Year Round	leaf	0.053	0.038	0.128	0.015	0.006	0.048	0.008	0.012	0.014	0.001	0.002	0.016
Lactuca sativa	lettuce	Butterhead	leaf	0.017	0.012	0.127	0.008	0.006	0.050	0.003	0.004	0.005	0.004	0.004	0.003
Lactuca sativa	lettuce	Little Gem	leaf	0.021	0.018	0.247	0.005	0.003	0.035	0.001	0.009	0.021	0.004	0.003	0.002
Lactuca sativa	lettuce	Rosetta	leaf	0.009	0.018	0.105	0.009	0.003	0.050	0.002	0.006	0.043	0.003	0.001	0.002
Lactuca serriola	wild lettuce	Serriola	leaf	0.027	0.029	0.280	0.011	0.002	0.047	0.002	0.002	0.018	0.001	0.001	0.007
Raphanus sativus	radish	Celesta	leaf	0.087	0.054	0.048	0.009	0.024	0.012	0.015	0.022	0.019	0.005	0.002	0.002
Raphanus sativus	radish	Expo	leaf	0.119	0.046	0.014	0.012	0.029	0.028	0.013	0.006	0.001	0.007	0.002	0.001
Raphanus sativus	radish	Sparkler	leaf	0.111	0.130	0.028	0.015	0.047	0.020	0.017	0.026	0.000	0.007	0.001	0.011
Spinacia oleracea	spinach	Amazon	leaf	0.285	0.310	0.309	0.013	0.011	0.097	0.054	0.041	0.022	0.003	0.007	0.034
Spinacia oleracea	spinach	Perpetual	leaf	0.582	0.176	0.322	0.167	0.133	0.530	0.031	0.049	0.007	0.014	0.015	0.067
Spinacia oleracea	spinach	Viking	leaf	0.206	0.073	0.050	0.018	0.017	0.328	0.019	0.003	0.008	0.003	0.001	0.068
Solanum lycopersicum	tomato	Alisa Craig	leaf	0.044	0.018	0.052	0.010	0.016	0.437	0.002	0.003	0.009	0.004	0.001	0.079
Solanum lycopersicum	tomato	Moneymaker	leaf	0.048	0.015	0.042	0.009	0.012	0.496	0.013	0.004	0.000	0.002	0.003	0.073
Hordeum vulgare	barley	Optic	root	0.250	0.079	0.045	0.098	0.001	0.061	0.058	0.060	0.040	0.090	0.001	0.016
Ocimum basilicum	basil	Gecofure	root	0.252	0.037	0.030	0.019	0.004	0.220	0.020	0.000	0.005	0.013	0.001	0.020
Lactuca sativa	lettuce	All Year Round	root	0.295	0.158	0.500	0.014	0.018	0.198	0.016	0.070	0.033	0.006	0.016	0.060
Lactuca sativa	lettuce	Butterhead	root	0.069	0.016	0.128	0.009	0.013	0.021	0.006	0.008	0.001	0.003	0.006	0.010
Lactuca sativa	lettuce	Little Gem	root	0.092	0.017	0.144	0.018	0.002	0.038	0.008	0.008	0.004	0.005	0.001	0.014
Lactuca sativa	lettuce	Rosetta	root	0.101	0.009	0.120	0.005	0.003	0.026	0.023	0.005	0.023	0.000	0.003	0.005
Lactuca serriola	wild lettuce	Serriola	root	0.109	0.104	0.201	0.072	0.013	0.126	0.026	0.029	0.048	0.008	0.005	0.107
Raphanus sativus	radish	Celesta	root	0.115	0.020	0.012	0.015	0.036	0.039	0.003	0.008	0.001	0.004	0.004	0.004

Raphanus sativus	radish	Expo	root	0.487	0.017	0.007	0.022	0.016	0.122	0.007	0.002	0.007	0.008	0.001	0.005
Raphanus sativus	radish	Sparkler	root	0.257	0.023	0.070	0.036	0.058	0.095	0.023	0.012	0.033	0.018	0.046	0.048
Spinacia oleracea	spinach	Amazon	root	0.161	0.026	0.707	0.006	0.012	0.010	0.023	0.023	0.071	0.006	0.010	0.005
Spinacia oleracea	spinach	Perpetual	root	0.326	0.062	0.102	0.032	0.032	0.434	0.014	0.003	0.000	0.011	0.002	0.002
Spinacia oleracea	spinach	Viking	root	0.154	0.033	0.060	0.041	0.029	0.369	0.014	0.013	0.022	0.013	0.013	0.104
Solanum lycopersicum	tomato	Alisa Craig	root	0.306	0.221	0.256	0.142	0.120	0.541	0.018	0.021	0.080	0.008	0.008	0.104
Solanum lycopersicum	tomato	Moneymaker	root	0.429	0.163	0.222	0.214	0.173	0.478	0.020	0.056	0.036	0.026	0.008	0.060
Brassica oleracea var. italica	broccoli*	Marathon	micro-leaf	0.354	0.014	0.008	0.038	0.009	0.421	0.075	0.000	0.001	0.003	0.004	0.086
Medicago sativa	alfalfa	not known	sprout	0.281	0.245	0.107	0.122	0.109	0.155	0.021	0.038	0.003	0.023	0.031	0.045
Trigonella foenum-graecum	fenugreek	not known	sprout	0.313	0.258	0.060	0.061	0.048	0.057	0.005	0.012	0.005	0.001	0.003	0.005

Pectin-enriched fractions (CDTA-treated, as per [2]) of horticultural and arable crop leaves and roots (barley, basil, lettuce, wild lettuce, radish, spinach, tomato), and sprouted seeds (alfalfa, fenugreek, prepared as per [3]) screened with selected antibodies (LM6, anti-(1→5)-α-L-arabinan (1 residue) [4]; LM13, anti-(1→5)-α-L-arabinan (3+ residues) [5]; LM5, anti-(1→4)-β-D-galactan [6]; LM16, Rhamnogalacturonan I epitope (uncharacterised) [5]; LM1, Extensin (histidine-rich glycoprotein) [7]; JIM13, β-D-GlcA-(1,3)-α-D-GalA-(1,2)-α-L-Rha (arabinogalactan protein) [8]). ELISA data is expressed as averaged absorbance (405 nm) in a heat-map format with standard deviations from replicated samples. ELISA data colour scale: high (red) to low (white). * broccoli microgreen data added from Fig. 5 for comparison, probed with the same antibodies except LM2 (1 → 6)-β-D galactan chain with terminally attached GlcA (arabinogalactan protein) [7, 8] was used in place of JIM13.

Supplementary References

1. Wang, R.F. and S.R. Kushner, Construction of versatile low-copy-number vectors for cloning, sequencing and gene expression in *Escherichia coli. Gene*, 1991. **100**: p. 195-9.
2. Rossez, Y., et al., *Escherichia coli* common pilus (ECP) targets arabinosyl residues in plant cell walls to mediate adhesion to fresh produce plants. *J Biol Chem*, 2014. **289**: p. 34349-34365.
3. Merget, B., et al., Influence of plant species, tissue type, and temperature on the capacity of Shiga-Toxigenic *Escherichia coli* to colonize, grow, and be internalized by plants. *Appl Environ Microbiol*, 2019. **85**(11).
4. Willats, W.G., S.E. Marcus, and J.P. Knox, Generation of monoclonal antibody specific to (1-5)-alpha-L-arabinan. *Carbohydr Res*, 1998. **308**(1-2): p. 149-52.
5. Verhertbruggen, Y., et al., Developmental complexity of arabinan polysaccharides and their processing in plant cell walls. *The Plant Journal*, 2009. **59**(3): p. 413-425.
6. Jones, L., G.B. Seymour, and J.P. Knox, Localization of pectic galactan in tomato cell walls using a monoclonal antibody specific to (1 [->] 4)-[b]-D-galactan. *Plant Physiol*, 1997. **113**(4): p. 1405-1412.
7. Smallwood, M., H. Martin, and J.P. Knox, An epitope of rice threonine-and hydroxyproline-rich glycoprotein is common to cell wall and hydrophobic plasma-membrane glycoproteins. *Planta*, 1995. **196**(3): p. 510-522.
8. Yates, E.A., et al., Characterization of carbohydrate structural features recognized by anti-arabinogalactan-protein monoclonal antibodies. *Glycobiol*, 1996. **6**(2): p. 131-9.

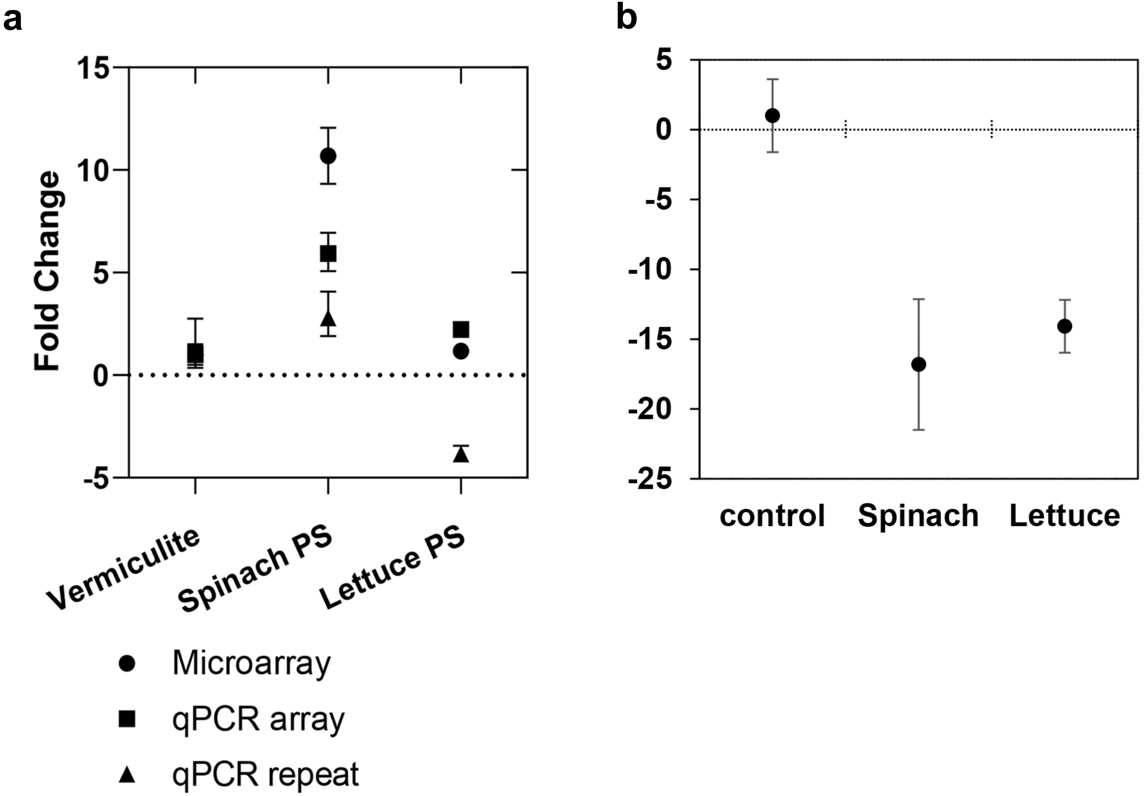
a
15
10
5
0
-5
Fold Change
Vermiculite
Spinach PS
Lettuce PS
Microarray
qPCR array
qPCR repeat
b
5
0
-5
-10
-15
-20
-25
control
Spinach
Lettuce

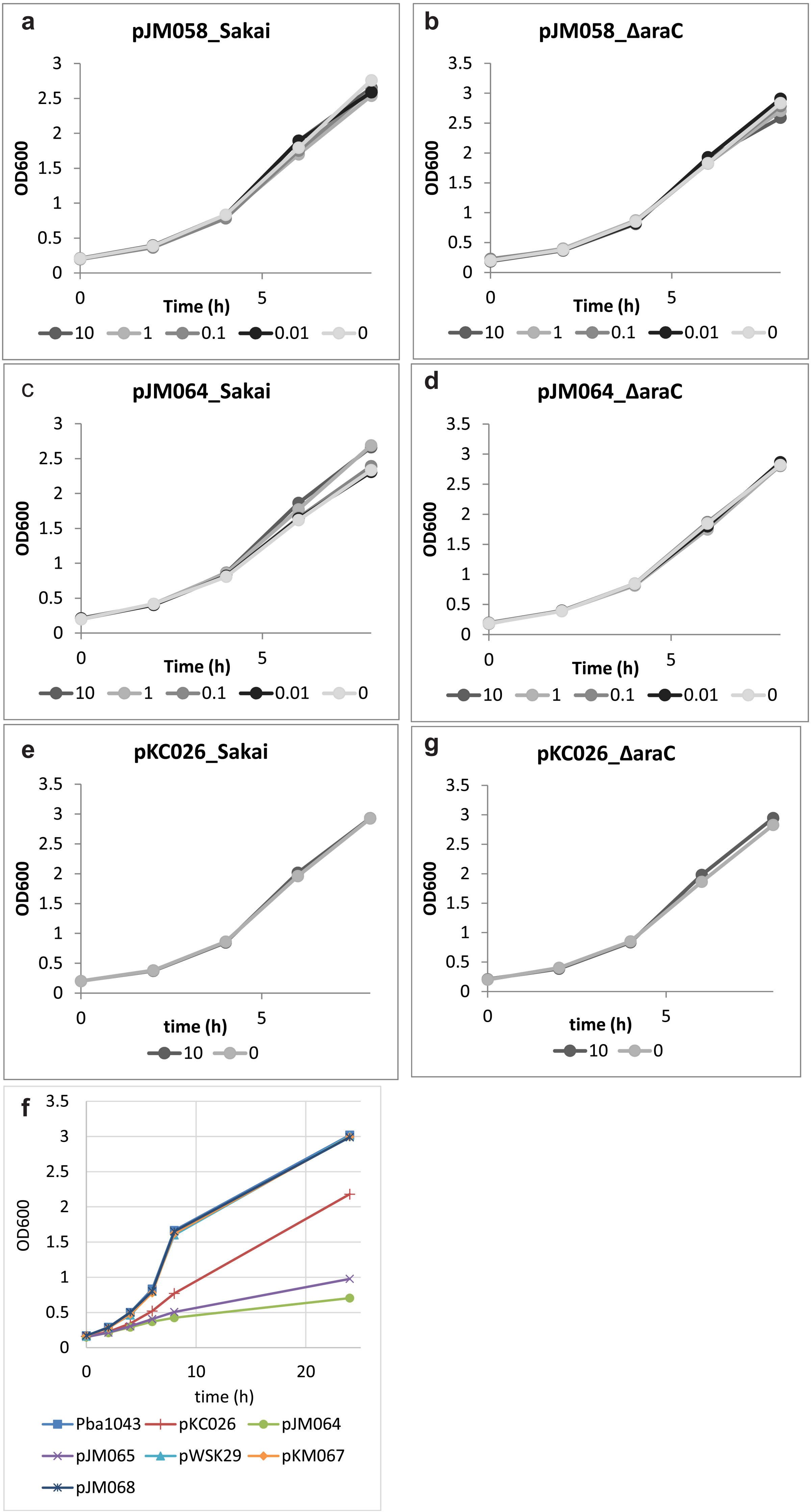

a
pJM058_Sakai
OD600
Time (h)
10 1 0.1 0.01 0
b
pJM058_ΔaraC
OD600
Time (h)
10 1 0.1 0.01 0
c
pJM064_Sakai
OD600
Time (h)
10 1 0.1 0.01 0
d
pJM064_ΔaraC
OD600
Time (h)
10 1 0.1 0.01 0
e
pKC026_Sakai
OD600
time (h)
10 0
g
pKC026_ΔaraC
OD600
time (h)
10 0
f
OD600
time (h)
Pba1043 pKC026 pJM064
pJM065 pWSK29 pKM067
pJM068

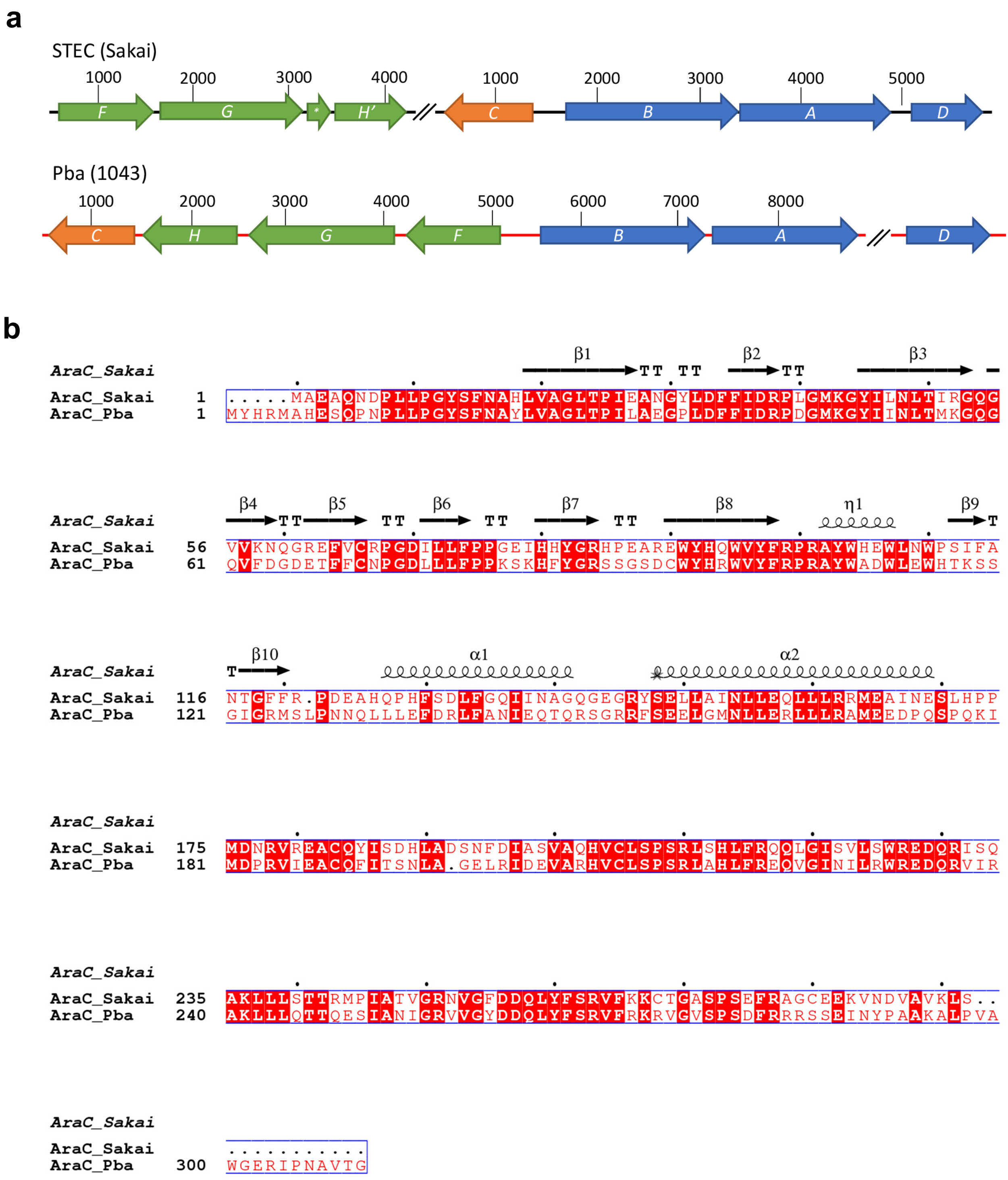
a
STEC (Sakai)
1000 2000 3000 4000 1000 2000 3000 4000 5000
F G * H' C B A D
Pba (1043)
1000 2000 3000 4000 5000 6000 7000 8000
C H G F B A D
b
β1 β2 β3 β4 β5 β6 β7 β8 η1 β9 β10 α1 α2
AraC_Sakai 1 .....MAEAQNDPLLPGYSFNAHLVAGLTPIEANGYLDFFIDRPLGMKGYILNLTIRGQG
AraC_Pba 1 MYHRMAHESQPNPLLPGYSFNAYLVAGLTPILAEGPLDFFIDRPDGMKGYIINLTMKGQG
AraC_Sakai 56 VVKNQGREFVCRPGDILLFPPGEIHHYGRHPEAREWYHQWVYFRPRAYWHEWLNWPSIFA
AraC_Pba 61 QVFDGDETFFCNPGDLLLFPPKSKHFYGRSSGSDCWYHRWVYFRPRAYWADWLEWHTKSS
AraC_Sakai 116 NTGFFR.PDEAHQPHFSDLFGQIINAGQGEGRYSELLAINLLEQLLLRRMEAINESLHPP
AraC_Pba 121 GIGRMSLPNNQLLLEFDRLFANIEQTQRSGRRFSEELGMNLLERLLLRAMEEDPQSPQKI
AraC_Sakai 175 MDNRVREACQYISDHLADSNFDIASVAQHVCLSPSRLSHLFRQQLGISVLSWREDQRISQ
AraC_Pba 181 MDPRVIEACQFITSNLA.GELRIDEVARHVCLSPSRLAHLFREQVGINILRWREDQRVIR
AraC_Sakai 235 AKLLLSTTRMPIATVGRNVGFDDQLYFSRVFKKCTGASPSEFRAGCEEKVNDVAVKLS..
AraC_Pba 240 AKLLLQTTQESIANIGRVVGYDDQLYFSRVFRKRVGVSPSDFRRRSSEINYPAAKALPVA
AraC_Sakai ....
AraC_Pba 300 WGERIPNAVTG

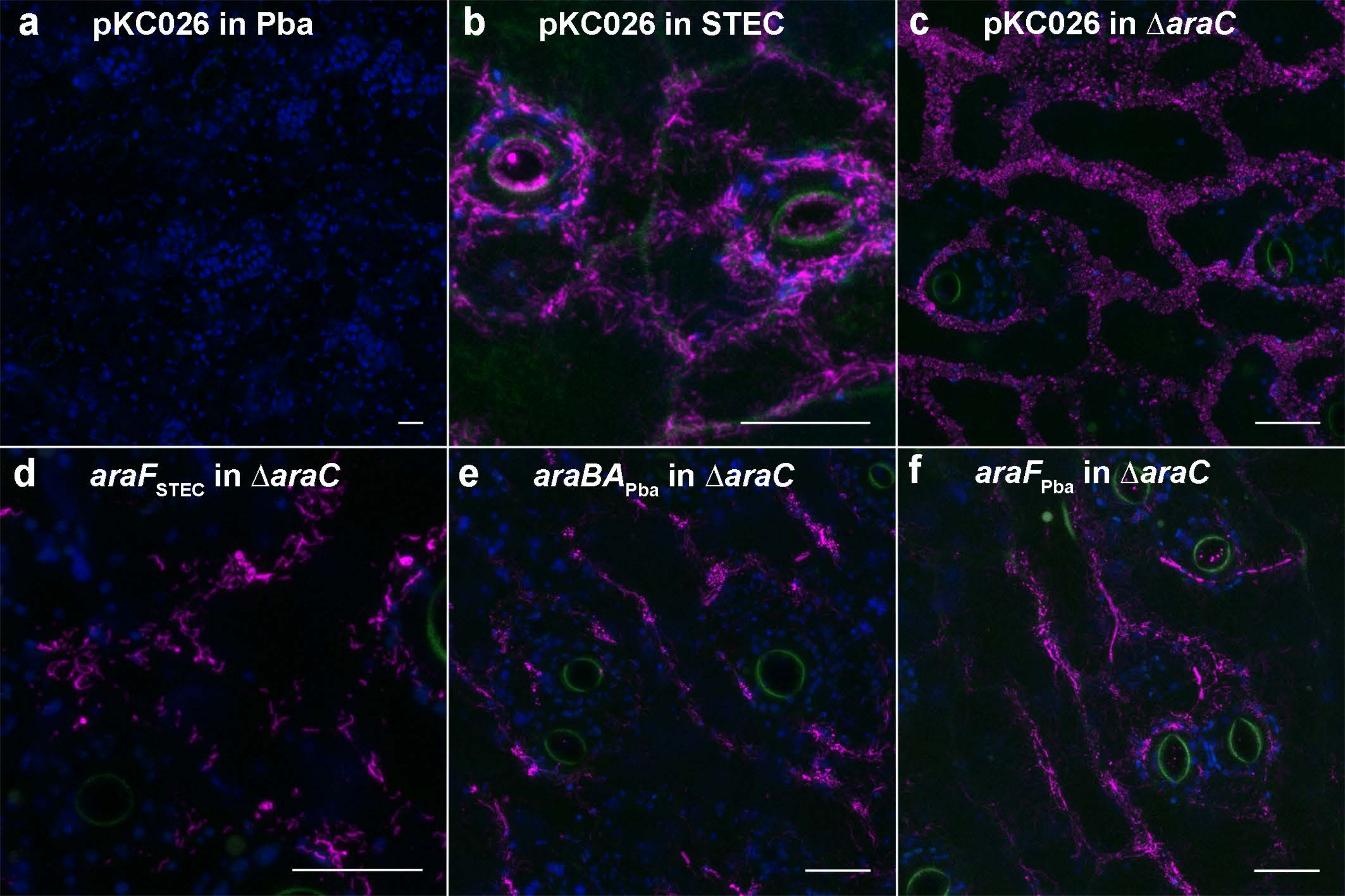

a pKC026 in Pba
b pKC026 in STEC
c pKC026 in ΔaraC
d araF STEC in ΔaraC
e araBA Pba in ΔaraC
f araF Pba in ΔaraC
